# Nicotinic Acetylcholine Receptor Subunit α7 Mediates Cigarette Smoke-Induced PD-L1 Expression in Human Bronchial Epithelial Cells

**DOI:** 10.3390/cancers13215345

**Published:** 2021-10-25

**Authors:** Hoi-Hin Kwok, Boning Gao, Koon-Ho Chan, Mary Sau-Man Ip, John Dorrance Minna, David Chi-Leung Lam

**Affiliations:** 1 Department of Medicine, Li Ka Shing Faculty of Medicine, The University of Hong Kong, Hong Kong, China; kwokh@hku.hk (H.-H.K.); koonho@hku.hk (K.-H.C.); msmip@hku.hk (M.S.-M.I.); 2 Hamon Center for Therapeutic Oncology Research, University of Texas Southwestern Medical Center, Dallas, TX 75390, USA; boning.gao@utsouthwestern.edu (B.G.); john.minna@utsouthwestern.edu (J.D.M.)

**Keywords:** lung cancer, cigarette smoke, PD-L1, *CHRNA7*, bronchial epithelial cells

## Abstract

**Simple Summary:**

Lung cancer is strongly associated with tobacco smoking. Nicotine in tobacco smoke can be transformed into a carcinogen after in vivo metabolism. However, the mechanism of how nicotine metabolites modulate a pro-tumorigenic microenvironment remains largely unknown. We have explored the effects of nicotine stimulation on nicotinic acetylcholine receptor subunit alpha 7 (nAChRα7)-mediated PD-L1 expression in human bronchial epithelial cells (HBECs). PD-L1 is an important protein contributing to cancer immune escape and is currently the key biomarker for lung cancer immunotherapy. Here, we found that the nicotine metabolite NNK can induce PD-L1 expression in HBECs by acting through the nicotine receptor nAChRα7. This study has a direct impact on the understanding of smoking-related changes in bronchial epithelial cells and potential immune escape in smoking-related carcinogenesis and has immuno-therapeutic implications via PD-L1 in lung cancer. Further studies are warranted to examine the upstream inhibition of PD-L1-related pathways, which could be mediated through nicotinic acetylcholine receptors in smokers, and may ultimately aid in the prevention and treatment of lung cancer.

**Abstract:**

Tobacco smoking is the top risk factor for lung cancer development. Nicotine in cigarettes can induce addiction, and its derivatives become potent carcinogens after metabolic activation and activate oncogenic signaling in lung epithelial cells through their expressed nicotinic acetylcholine receptors (nAChRs). However, the effects of smoking on the tumor immune microenvironment are under investigation. In the current study, we investigated whether nicotine activation of nicotinic acetylcholine receptor subunit α7 (nAChRα7, *CHRNA7*) would induce PD-L1 expression in lung epithelial cells. The expression levels of nAChRα7 and PD-L1 in eight human bronchial epithelial cell (HBEC) lines were measured after treatment with cigarette smoke extract (CSE) or nicotine derivatives. The results showed that PD-L1 expression levels increased in HBECs after exposure to CSE or nicotine derivatives. This induction of PD-L1 expression could be diminished by treatment with *CHRNA7* small-interfering RNA, and the relevant signaling was mediated via STAT3 phosphorylation and NRF2 expression. In summary, this study demonstrated that the well-known nicotine derivative-activated nAChRα7 could induce STAT3/NRF2 pathways and subsequently promote PD-L1 expression in normal lung epithelial cells. This information provides mechanistic insight into cigarette smoke-induced immune evasion in lung epithelial cells.

## 1. Introduction

Tobacco smoking is the top risk factor for the development of nearly all types of lung cancer. Among the different histological subtypes of lung cancer [1], squamous cell carcinoma (SCC) is strongly associated with tobacco smoking [2]. Many different types of carcinogens are found in cigarette smoke. While nicotine itself is not considered to be directly carcinogenic, addiction to nicotine is the major factor driving cigarette smoking and thus exposure to tobacco carcinogens. In addition, nicotine can be converted to 4-(methylnitrosoamino)-1-(3-pyridyl)-1-butanone (NNK), which is a potent carcinogen from metabolic activation. This role is demonstrated by how potent NNK is in inducing lung tumor development in animal models [3]. As part of this, NNK metabolites bind to DNA, forming DNA adducts which can result in genetic mutations.

In previous genome-wide association studies (GWASs), genetic polymorphisms in chromosome region 15q25 were found to be associated with increased risk of developing lung cancer. This 15q25 region harbors three nicotinic acetylcholine receptor (nAChR) subunit genes, namely *CHRNA3*, *CHRNA4*, and *CHRNA5* [4]. These genetic variants may affect nicotine dependence, leading to greater exposure or susceptibility to carcinogens in cigarette smoke and hence increased risk of lung cancer development [5]. In fact, *CHRNA7* (located at 15q13.3) gene duplication was found to increase lung cancer risk [6]. In addition, several studies showed that lung epithelial cells and lung cancer can express nAChRs and that nicotine at concentrations found in smokers can bind to these receptors and activate signaling mechanisms that could play a role in lung cancer pathogenesis [7]. These include studies that showed that cigarette smoking can act through nAChRα7 to modulate inflammatory responses [8]. All these studies suggested that nicotine-based ligand-activated nAChR and its downstream signaling aberrations may contribute to lung cancer development.

The use of immune checkpoint inhibitor/blockade (ICB) therapy has emerged as an important treatment strategy for NSCLC in recent years. The best treatment response was shown in patients with high tumor expression levels of programmed death receptor ligand-1 (PD-L1), usually with a tumor proportional score (TPS, % of tumor cells expressing PD-L1) above 50%. Smokers with lung cancer are usually seen to derive more benefit from immune checkpoint therapy compared to non-smokers with lung cancer [9]. Smoking status was significantly associated with tumor mutation burden [10] and with higher tumor PD-L1 expression [11] in lung cancer. However, the correlation of PD-L1 expression and tumor mutation burden with clinical benefit from ICB is yet to be determined. There is also consideration of employing ICB to intercept pre-neoplastic lesions. The molecular mechanisms regulating PD-L1 expression in lung cancer and the tumor microenvironment are being explored, and recent functional genomic approaches have identified several unexpected mechanisms [12]. Our previous studies demonstrated that nicotine could stimulate *CHRNA7* expression in human bronchial epithelial cells (HBECs) and that HBECs could be used to study the pathogenesis of lung cancer through defined genetic manipulations [7,13]. The aim of this study was to test whether nicotine and cigarette smoke extract (CSE) could act through defined nAChRα7 to influence the expression of PD-L1 in HBECs.

## 2. Materials and Methods

### 2.1. In Silico Gene Expression Analysis

Gene expression data of different *CHRN* subunit genes in normal lung were downloaded from Genotype-Tissue Expression (GTEx). Differential expression analysis of *CHRN* subunit genes between normal and lung tumor lungs was performed using Lung Cancer Explorer [14] and UALCAN [15].

### 2.2. Cell Culture

Eight immortalized human bronchial epithelial cell (HBEC) lines were used. These cell lines were established from bronchial biopsies obtained during diagnostic bronchoscopy. HKBS62N-KT, HKBS65.2N-KT, HKBS160N-KT, and HKBS189N-KT were derived from smokers; HKBS150N-KT, HKBS197N-KT, HKBS198N-KT, and HKBS202.5N-KT were derived from non-smokers. Bronchial specimens from local non-cancer patients were obtained with informed consent. These subjects had bronchoscopy with autofluorescence imaging performed, and the bronchial biopsies were all confirmed histologically to be non-neoplastic [7]. In bronchoscopy with autofluorescence imaging (AFI), suspicious areas will show up as areas of magenta fluorescence color, in contrast to the green fluorescence color for normal bronchial epithelium. Bronchial biopsies of the normal green fluorescence color were cultured and immortalized to become cell lines used in this study. Immortalization of HBECs was performed according to the previously published protocol [16]. These cells were cultured in keratinocyte-serum-free medium and were treated with cigarette smoke extract (CSE, aqueous extract of two Marlboro cigarettes (12 mg tar, 1 mg nicotine) bubbled into 20 mL PBS, 0.22 μm filtered) [17] or NNK (#N-076, Sigma-Aldrich, St. Louis MO, USA) (5 or 10 μM) [18] in supplement-free medium as previously described [19].

### 2.3. RNA Extraction and Quantitation of mRNA Expression

Total RNA was extracted from HBECs using an RNAeasy mini extraction kit (#74106, Qiagen, Hilden, Germany). Complementary DNA was synthesized with the QuantiTect reverse transcription kit (#205311, Qiagen, Germany). The expression level of the target genes was determined by quantitative real-time PCR using the QuantiTect SYBR Green PCR kit (#204143, Qiagen, Germany). All the reactions were performed with a LightCycler480 II real-time PCR machine (Roche Diagnostics, Penzberg, Germany). The relative expression levels of *CHRNA7* subunit genes were normalized to the expression levels of *GAPDH*. The sequences of primers used were as follows: *CHRNA7*-F, 5′-AGC CAG CAA TTC TGA GTT CTG-3′; *CHRNA7*-R, 5′-TTG CCC ATC TCC AGT GAA TC-3′; *CD274*-F, 5′- GGC ATC CAA GAT ACA AAC TCA AAG-3′; *CD274*-R, 5′-CTT CCT CTT GTC ACG CTC AG-3′; *GAPDH*-F, 5′-GTC AGT GGT GGA CCT GAC CT-3′; *GAPDH*-R, 5′-TGA GCT TGA CAA AGT GGT CG-3′.

### 2.4. Protein Extraction and Immunoblot Analysis

Whole cell protein was extracted using RIPA buffer (#89900, Thermo Fisher, San Jose, CA, USA) supplemented with protease and phosphatase inhibitor (#A32959, Thermo Fisher, San Jose, CA, USA). The protein concentration of the cell lysate was determined by protein assay (#5000006, Bio-Rad, Hercules, CA, USA). Protein samples were separated by 10% SDS-PAGE and blotted onto nitrocellulose membranes (#1228243, Santa Cruz Biotechnology, Santa Cruz, CA, USA). The membranes were then incubated in blocking buffer (5% non-fat dry milk in TBS-T) and subsequently in diluted primary antibodies against specific targets at 4 °C overnight. The membranes were then washed and incubated with horseradish peroxidase-conjugated goat anti-rabbit or goat anti-mouse secondary antibody (#7074 and #7076, Cell Signaling Technology, Cell Signaling Technology, Danvers, MA, USA). Immunoreactive bands were visualized by ECL Western blotting reagents (#RPN2106, GE Healthcare, Chalfont St Giles, IJ, UK). Primary antibodies, including anti-nAChRα7 (#sc-5544, 1:500, Santa Cruz Biotechnology, USA), anti-PD-L1 (#sc-50298, 1:500, Santa Cruz Biotechnology, USA), anti-STAT3 (#9139, 1:1000, Cell Signaling Technology, USA), anti-phospho-STAT3 (Y705) (#9145, 1:500, Cell Signaling Technology, USA), anti-NRF2 (#16396-1-AP, 1:500, Proteintech, Rosemont, IL, USA), and HRP-conjugated anti-GAPDH (#HRP-60004, 1:2000, Proteintech, USA), were used in the immunoblot analysis.

### 2.5. Immunofluorescence Microscopy

HBECs (1 × 10^4^ cells/well) were plated onto a 96-well cell culture plate overnight. After NNK treatment, cells were fixed in cold paraformaldehyde (4%, *w*/*v*) at room temperature for 15 min. After washing with PBS three times, cells were incubated in blocking reagent (1% BSA in PBS) for one hour and then in primary anti-PD-L1 (#sc-50298, 1:200, Santa Cruz Biotechnology, USA) antibody at 4 °C overnight. Cells were washed with PBS and then incubated with Alexa Fluor 488-conjugated goat anti-rabbit secondary antibody (#A-11008, 1:500, Thermo Fisher, USA) at room temperature for one hour. Nuclei were counter-stained with DAPI (0.5 μg/mL). Plates were washed with PBS three times, and fluorescence images were acquired using the ImageXpress Pico Automated Cell Imaging System (Molecular Devices, Union City, CA, USA) with a 10× objective lens. Positive cell-integrated intensities were used to compare the fluorescence images from different treatments.

### 2.6. Transient Transfection of Small Interfering RNA (siRNA)

HBECs at 60% confluence were transiently transfected with *CHRNA7*-specific siRNA (#S103054443, FlexiTube siRNA) or non-targeting control siRNA (#1027281, AllStars Negative Control) (20 nM) obtained from Qiagen. Cells were treated with an RNA–lipofectamine complex (Lipofectamine 3000, #L3000015, Thermo Fisher, USA) in supplement-free medium for 24 h followed by NNK treatment.

### 2.7. Cignal 45-Pathway Reporter Arrays

Cignal 45-pathway reporter arrays were used according to the manufacturer’s protocol. Briefly, 96-well plates coated with designated reporter plasmids were mixed with transfection reagent (Lipofectamine 3000, #L3000015, Thermo Fisher, USA). HBECs transfected with siRNA (1 × 10^4^ cells/well) were then transferred to the 96-well plates. The transfection medium was removed after 24 h, and cells were then treated with NNK (10 μM) for another 24 h. Luciferase activity was detected using a dual-luciferase reporter assay system (#E1960, Promega, Madison, WA, USA). The luminescence signal was then measured using a microplate reader (CLARIOstar BMG Labtech, Ortenberg, Germany). The firefly luciferase activity was normalized by the Renilla luciferase activity in each well.

### 2.8. Statistical Analyses

*CHRNA7* levels are reported as mean ± standard deviation. Comparisons between different treatment groups were analyzed by either chi-square tests or Student’s *t*-tests. Multiple group comparisons were conducted with one-way ANOVA followed by Tukey’s post hoc analyses. GraphPad Prism version 6.01 (GraphPad Software Inc., La Jolla, CA, USA) was used for all statistical analyses. A two-sided *p*-value of less than 0.05 was considered statistically significant.

## 3. Results

### 3.1. CHRNA7 Expression Is Associated with Lung Cancer

**Figure 1:**
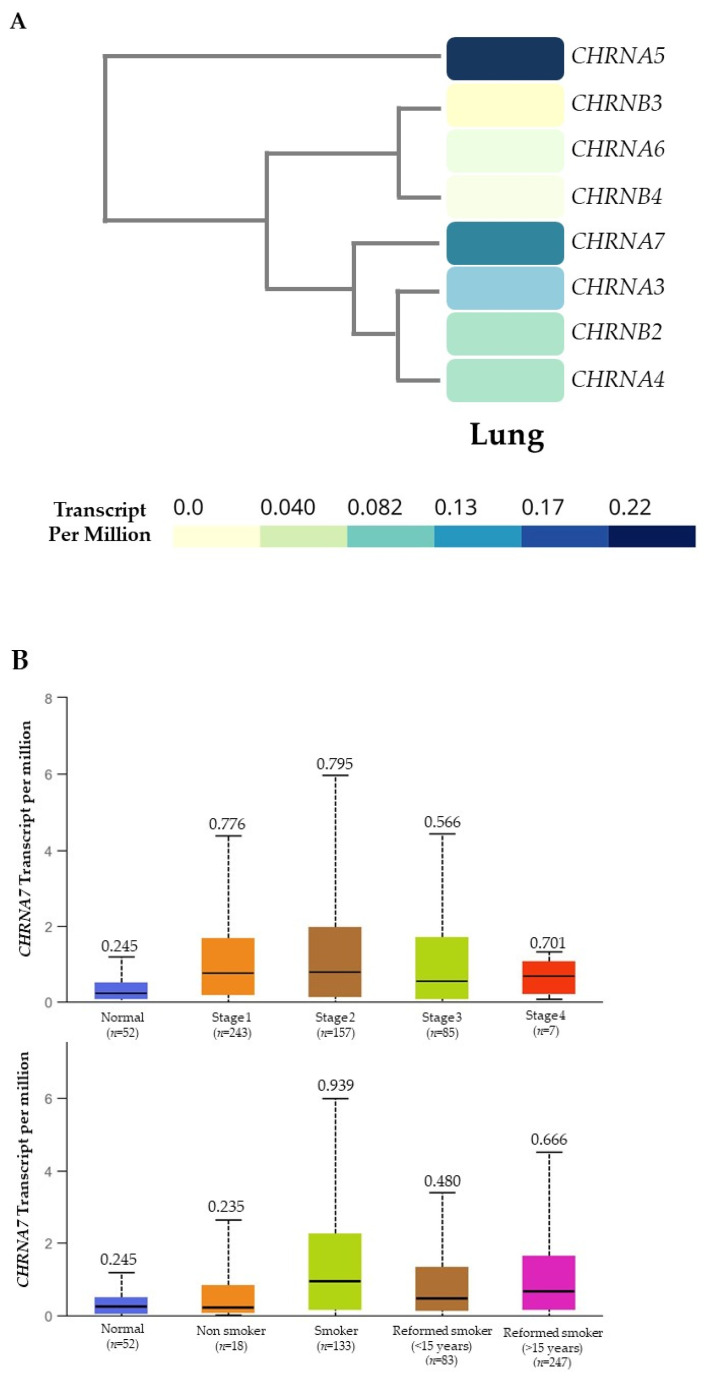

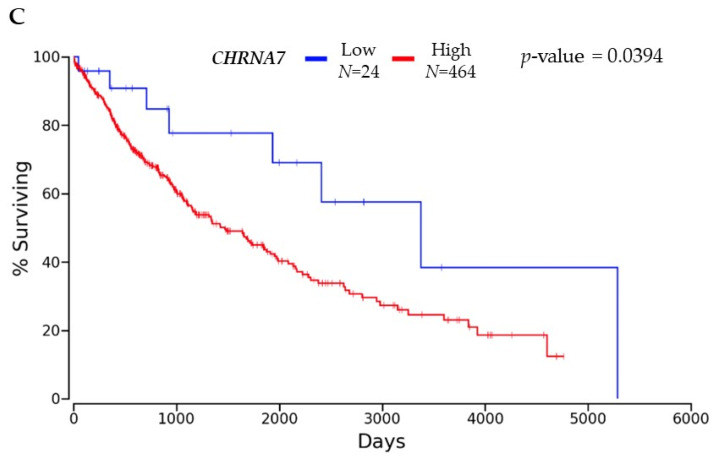
*CHRNA7* expression and clinicopathological correlations in TCGA-LUSC cohort. (**A**) Expression levels of *CHRN* subunit genes in normal lung from GTEx portal. (**B**) Boxplots of *CHRNA7* gene expression comparing normal lung tissues against different stages of lung cancer (upper panel), or non-smoking patients against patients with different smoking habits (lower panel) in the TCGA-LUSC dataset; median of each bar is shown. (**C**) Kaplan–Meier analysis of lung squamous cell carcinoma patients in the TCGA-LUSC cohort (Oncolnc).

The *in silico* gene expression analysis showed that *CHRNA5* and *CHRNA7* were the two most highly expressed *CHRN* subunit genes in the normal lung tissues (Figure 1A). Moreover, *CHRNA7* was overexpressed in lungs with cancer compared to non-cancer-involved “normal” lungs by over 30% (Figure 1B, upper panel). *CHRNA7* expression levels were also higher in smokers than in non-smokers (Figure 1B, lower panel). In fact, the higher expression of *CHRNA7* in lung squamous cell carcinoma was associated with poor survival in the TCGA-LUSC cohort (Figure 1C).

### 3.2. nAChRα7-Mediated Smoking-Induced PD-L1 Expression in HBECs

**Figure 2:**
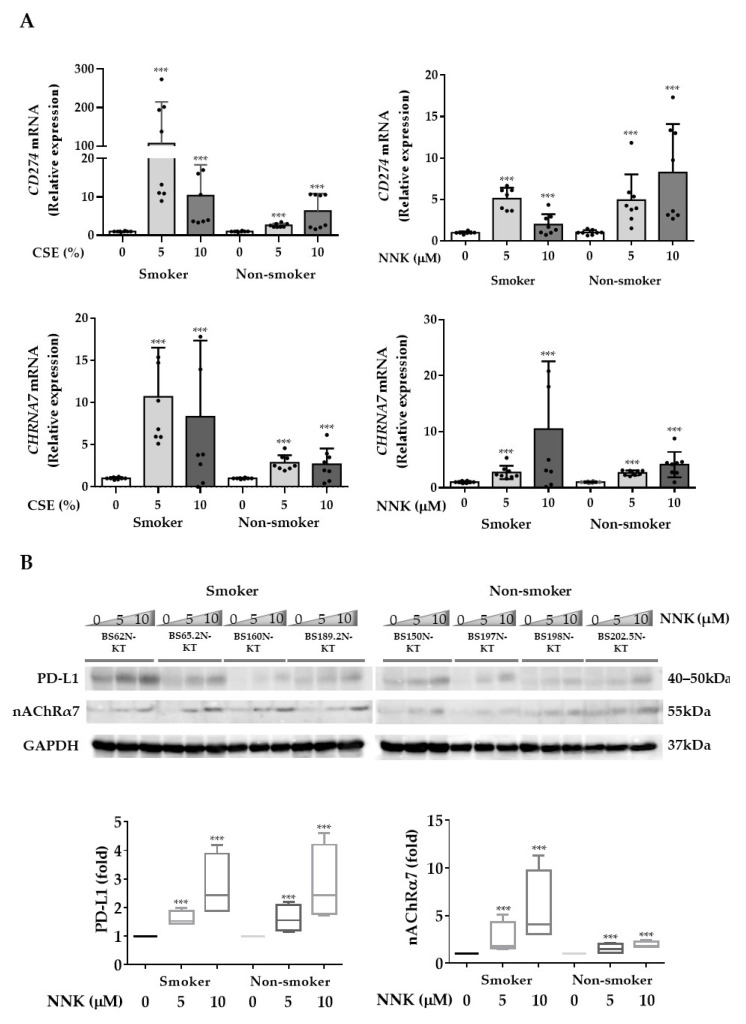

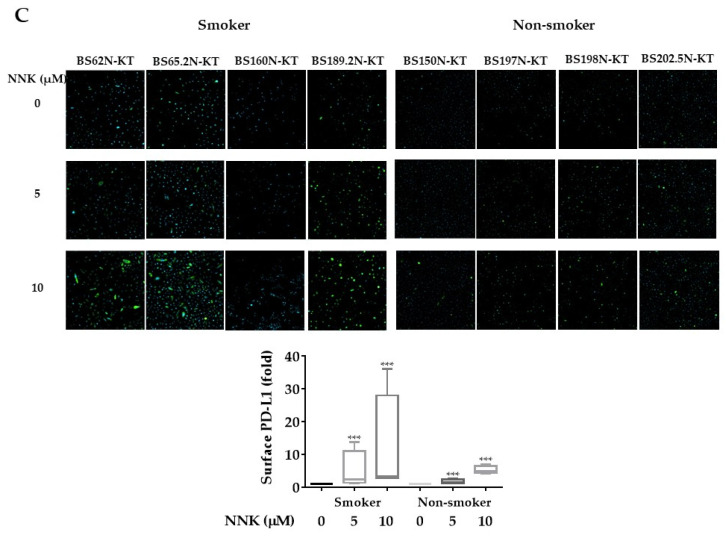
Cigarette smoke and the carcinogen NNK induce PD-L1 expression on human bronchial epithelial cells (HBECs). HBECs derived from smokers (BS62N-KT, BS65.2N-KT, BS160N-KT, and BS189.2N-KT) or non-smokers (BS150N-KT, BS197N-KT, BS198N-KT, and BS202.5N-KT) were exposed to cigarette smoke extract (CSE, 5 or 10%) or NNK (5 or 10 µM) for 48 h, and *CD274* and *CHRNA7* (**A**) mRNA expression was measured by real-time RT-PCR; (**B**) PD-L1 and nAChRα7 protein expression was determined by Western blot analysis; (**C**) surface PD-L1 expression was measured by immunofluorescence staining. The values are presented as mean ± S.D. from technical triplicate experiments. *** *p* < 0.001.

Both CSE and NNK were found to be able to induce *CHRNA7* and PD-L1 (*CD274*) mRNA expression in HBECs derived from both smokers and non-smokers (Figure 2A). Furthermore, the immunoblot analysis showed that NNK could induce nAChRα7 and PD-L1 protein expression in HBECs (Figure 2B and Figure S2) and their expression on the cell surface by immunofluorescence staining (Figure 2C).

**Figure 3:**
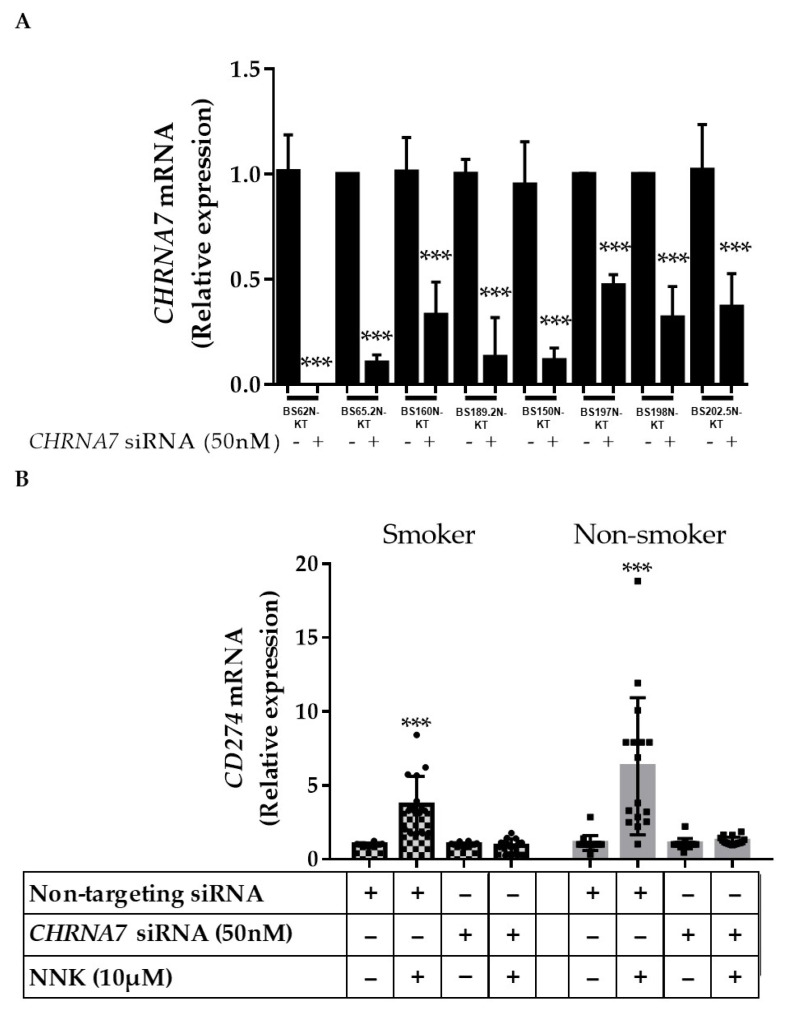

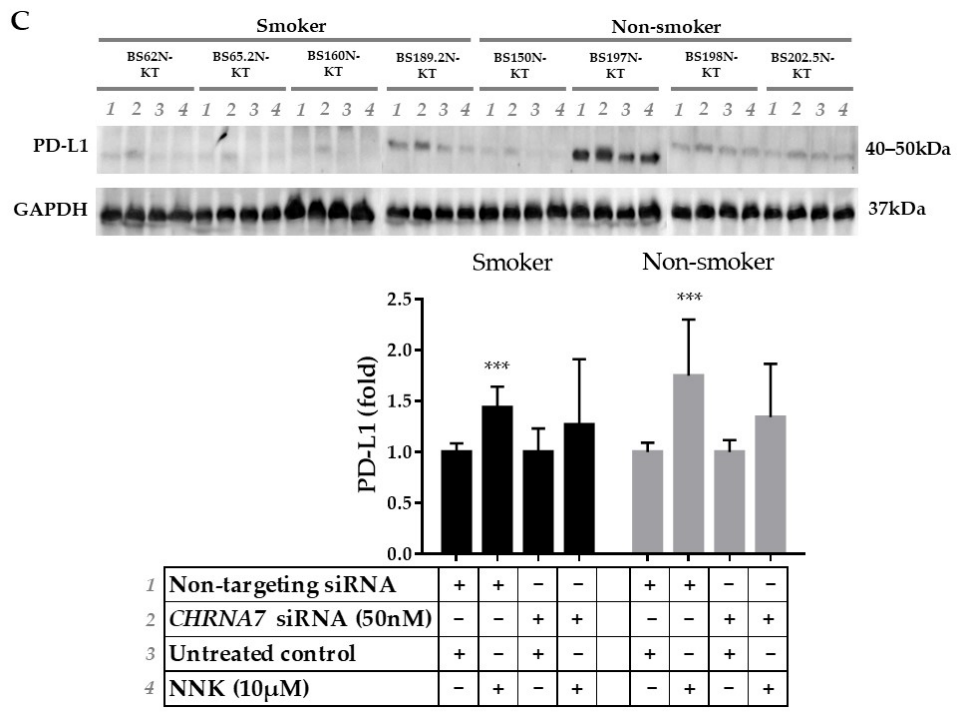
nAChRα7-mediated NNK-induced PD-L1 expression. (**A**) HBECs were treated with non-targeting or *CHRNA7*-specific siRNA (50 nM) for 24 h, and the expression of *CHRNA7* mRNA was measured by real-time RT-PCR. HBECs with or without knockdown of *CHRNA7* by siRNA (50 nM) for 24 h were further treated with NNK (10 μM) for another 48 h; the expression of *CD274* (PD-L1) (**B**) mRNA expression was measured by real-time RT-PCR; (**C**) protein expression was determined by Western blot analysis. The values are presented as mean ± S.D. from technical triplicate experiments. *** *p* < 0.001.

To determine if NNK was acting through nAChRα7 to induce PD-L1 expression, we used siRNA-mediated knockdown of *CHRNA7* (Figure 3A). We saw that the inducing effects of NNK on PD-L1 mRNA (Figure 3B) and protein (Figure 3C and Figure S2) expression were abolished by *CHRNA7* knockdown in HBECs established from both smokers and non-smokers. This supports the role of nAChRα7 as a key mediator for NNK-induced PD-L1 expression. 

### 3.3. Antioxidant and STAT3 Pathways Were Involved in Mediating nAChRα7-Regulated PD-L1 Expression

**Figure 4:**
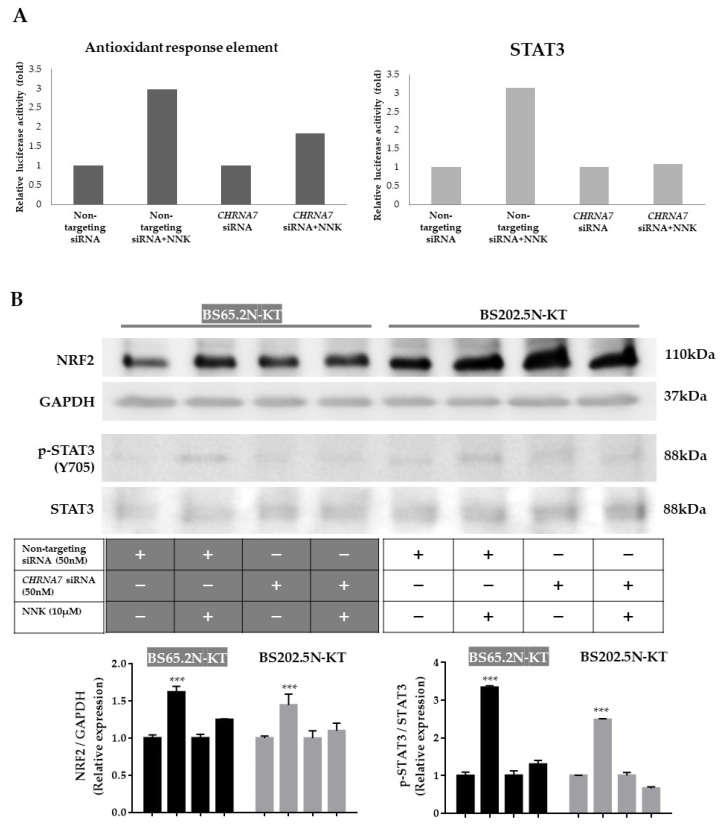

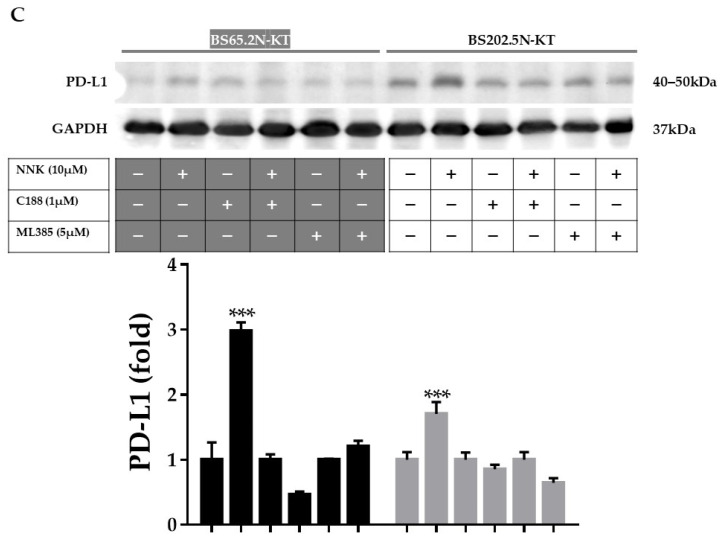
nAChRα7-mediated NNK-induced PD-L1 expression through NRF2 and STAT3 signaling. (**A**) HBECs transfected with non-targeting or *CHRNA7*-targeting siRNA (50 nM) for 24 h were seeded on the assay plate of a Cignal 45-pathway reporter array and then reverse-transfected with reporter plasmids. Cells were then treated with NNK (10 µM) for another 24 h. Firefly and Renilla luciferase activities were measured. Normalized luciferase activities were compared with those of non-NNK-treated cells. Representative pathways are shown. (**B**) HBECs were treated with non-targeting or *CHRNA7*-targeting siRNA (50 nM) for 24 h and then treated with NNK (10 µM) for another 48 h. The expression of NRF2 and STAT3 phosphorylation was measured with Western blot analysis. (**C**) HBECs were co-treated with NNK (10 µM) and the STAT3 inhibitor C188 (1 µM) or the NRF2 inhibitor ML385 (5 µM) for 48 h. PD-L1 protein expression levels were determined by Western blot analysis. The values are presented as mean ± S.D. from technical triplicate experiments. *** *p* < 0.001.

To explore the mechanisms by which nAChRα7 regulates PD-L1 expression, we performed promoter activity screening assays with the Cignal 45-pathway reporter array. To obtain NNK acting through nAChRα7 receptor-specific effects, we compared Cignal45-pathway reporter activity (luciferase activity) under four conditions: pathway activity after treatment with a non-*CHRNA7*-targeting siRNA (baseline activity after siRNA treatment); the same non-targeting siRNA but with NNK (activity seen with NNK acting through the receptor); specific knockdown of *CHRNA7* expression with a targeted siRNA without NNK; and knockdown of *CHRNA7* with NNK treatment (Supplementary Figure S1). These studies showed that NNK specifically acts through nAChRα7, activated DNA damage, *MYC, Wnt, NFE2L2*-antioxidant response elements, *STAT3*, and some other signaling pathways (Supplementary Figure S1). Knockdown of *CHRNA7* reversed some of these activations, particularly the *NFE2L2*-antioxidant response and *STAT3* signaling pathways (Figure 4A), suggesting the involvement of the two pathways in NNK-induced PD-L1 expression [20,21].

To investigate the roles of the NRF2 and STAT3 signaling pathways, Western blot analysis was performed to evaluate the induction or activation of NRF2 and STAT3 following NNK exposure. NNK treatment induced NRF2 protein expression and STAT3 phosphorylation in the two HBEC cell lines (Figure 4B and Figure S2), while siRNA-mediated *CHRNA7* knockdown abolished these effects. In addition, we pharmacologically confirmed that NRF2 and STAT3 were downstream mediators of NNK-induced PD-L1 expression by pretreating two HBECs with either the STAT3 inhibitor (C188-9) or the NRF2 inhibitor (ML385) prior to NNK treatment. Both inhibitors suppressed NNK-induced PD-L1 expression (Figure 4C and Figure S2) in the HBECs.

## 4. Discussion

By treating HBEC strains derived from different individuals with NNK, combined with studies on the expression and functional genomic knockdown of *CHRNA7*, we found that exposure of HBECs to CSE or NNK acting through nAChRα7 leads to significantly increased expression of PD-L1 on HBECs. In addition, we have provided evidence that this involves signaling through STAT3/NRF2. These findings provide a mechanism for the observed increased PD-L1 expression in bronchial epithelial tissues and SCC from smokers. The effects of *CHRNA7* knockdown on PD-L1 expression may also identify a chemopreventive opportunity to counteract the carcinogenic effects of tobacco smoking even after smoking cessation.

Recent studies suggested that different chemical substances, such as polycyclic aromatic hydrocarbons, aldehydes, various volatile hydrocarbons [22], and nicotine, which may also be present in electronic cigarette smoke could contribute to lung carcinogenesis [14]. It has been well demonstrated that nicotine derivatives, including NNK and NNN, can induce the formation of DNA adducts and initiate carcinogenesis. Nicotine and its derivatives can also bind with nAChRs and induce various aberrant cellular signaling processes involved in tumorigenesis. The in silico analysis (Figure 1) also suggested that smoking may induce higher expression of nAChRα7, which may contribute to cancer development via unknown mechanisms. Studies in different cancers suggested that PD-L1 expression was up-regulated in pre-malignant lesions [23,24], and early PD-L1 blockade may prevent cancer development [25]. However, whether nicotine and its derivatives could contribute to pre-malignant immune invasion through PD-L1 expression remained unknown. In this study, we confirmed the involvement of nAChRα7 mediation in nicotine derivative-induced PD-L1 expression in HBECs.

nAChRα7 is widely expressed in neuronal tissue, the digestive tract, and in the lungs and the airways; plays various roles in smoking-related schizophrenia [26] and chronic pulmonary diseases [6]; and is also associated with higher angiotensin-converting enzyme II (ACE-2) expression and subsequent higher risk of severe COVID-19 infection [27,28]. It is known that nAChRα7-mediated signaling is involved in the regulation of various tumorigenic processes [29]. In melanoma, it has been shown that nicotine could induce PD-L1 expression via *CHRNA9* and could promote cell proliferation and migration [30]. However, our study demonstrated, for the first time, a direct mediating role of nAChRα7 in smoking-induced PD-L1 expression in normal lung epithelial cells.

Transcriptional regulation of PD-L1 has been shown to be activated by various intrinsic oncogenic pathways. Cancer cells utilize interferon-activated JAK-STAT-IRF1 pro-inflammatory signaling to induce PD-L1 expression in cancer cells [31]. Moreover, dysregulation of *NRF2* could directly [32] or indirectly [33] increase PD-L1 expression levels. Cigarette smoke affects multiple intracellular responses in bronchial epithelial cells, including the above-mentioned pathways (Figure 4A). Nicotinic activation of *CHRNA7* promoted the phosphorylation of STAT3 and NRF2 expression (Figure 4B,C), which may synergistically promote the transcription of PD-L1. Previous studies suggested that smoking or oncogenes could induce persistent activation or overexpression of NRF2, which may in turn contribute to lung tumorigenesis [20,21]. Our study demonstrated the link of nAChRα7-activated STAT3/NRF2 pathways with downstream PD-L1 expression.

PD-L1 expression in normal tissues is tightly regulated by multiple signaling pathways to maintain immune homeostasis. Wang et al. previously demonstrated that benzo(α)pyrene from tobacco could induce PD-L1 expression in HBECs via the induction of aryl hydrocarbon receptor (AhR) [34]. The results of this study further support that various carcinogenic components in cigarette smoke may contribute to increased PD-L1 expression in normal bronchial epithelial cells. Gene Set Enrichment Analysis and Ingenuity Pathway Analysis on lung squamous cell carcinoma suggested a more pro-inflammatory phenotype in heavy smokers [35]. This may further promote pre-cancerous immune evasion, which may ultimately lead to cancer formation. Indeed, there are several ongoing clinical trials to investigate the possibility of prevention of malignant transformation by treating high-risk smokers with a PD-L1 inhibitor. Further studies are warranted to examine the upstream inhibition of PD-L1-related pathways, which could be mediated through nicotinic acetylcholine receptors in smokers. However, direct systemic administration of most nicotinic acetylcholine receptor antagonists can cause a variety of side effects due to the lack of nAChR subtype specificity [36]. Site-specific and subtype-specific inhibition of nAChRα7 may be able to address the controversial effects of nAChRα7 on tumor progression in different tissues [37].

## 5. Conclusions

In summary, this study demonstrated that cigarette smoke could induce PD-L1 expression on HBECs through the activation of nAChRα7 followed by activation of the STAT3 and NRF2 pathways. This study paves the way for further study on the role of specific immune-sensitizers that could aid the therapeutic effects of anti-PD-1 treatment in lung cancer.

## Data Availability

The authors declare that the data supporting the findings of this study are available within the article and its supplementary information files.

## Supplementary Materials

The following are available online at https://www.mdpi.com/article/10.3390/cancers13215345/s1. Figure S1: Waterfall plot of Cignal 45-pathway reporter array. Figure S2: Uncropped images for all Western blot analysis with the indicated target proteins.
