# Supplementary material for: Nicotinic Acetylcholine Receptor Subunit α7 Mediates Cigarette Smoke-Induced PD-L1 Expression in Human Bronchial Epithelial Cells
Source: Cancers (Basel). 2021 Oct 25;13(21):5345. doi: 10.3390/cancers13215345 (PMC8582493; doi:10.3390/cancers13215345)
Supplement: Supplementary file 1 [file cancers-13-05345-s001.zip › cancers-1417260-supplementary-final2.pdf]

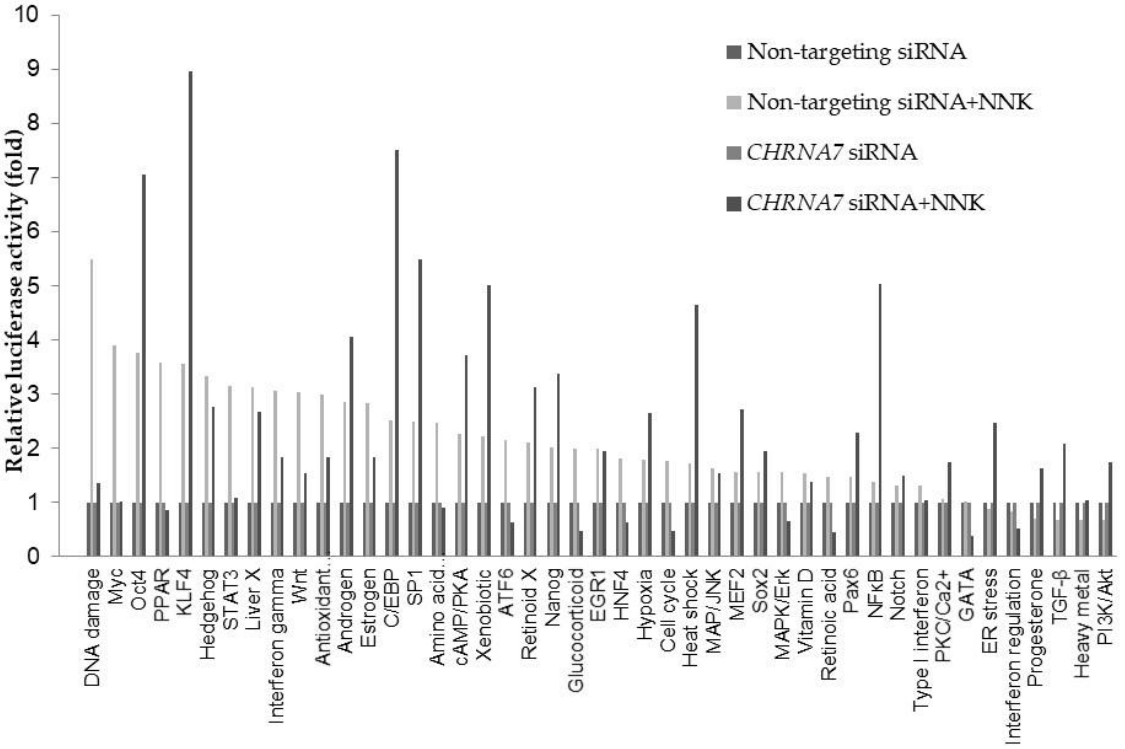

Figure S1. Waterfall plot of Cignal 45-pathway reporter array. BS65.2N-KT HBECs were transfected with *CHRNA7* siRNA or non-targeting siRNA and treated with or without NNK (10μM). Cells were then co-transfected with various luciferase reporter vectors included in the Cignal 45-pathway reporter array. Normalized luciferase activities were compared with those of NNK-untreated cells.

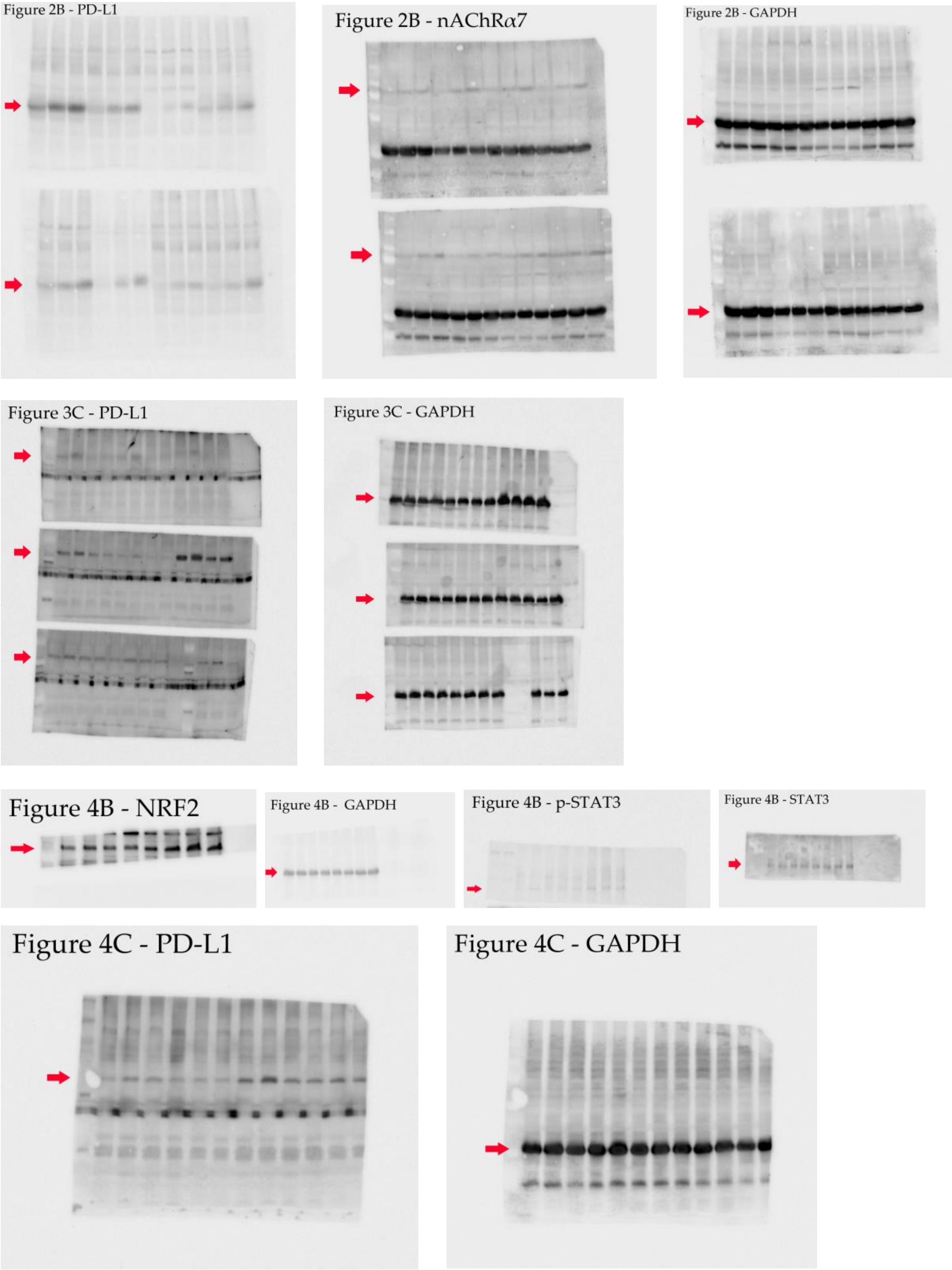

Figure S2. Uncropped images for all Western blot analysis with the indicated target proteins.
